# Neuralgia of the Ovaries, Treated with the Muriate of Amonia and Tr. of Aconite

**Published:** 1869-11

**Authors:** James T. Newman

**Affiliations:** Chicago


### ARTICLE XL.

### NEURALGIA OF THE OVARIES, TREATED WITH THE MURIATE OF AMONIA AND TR. OF ACONITE.

BY JAMES T. NEWMAN, M.D., Chicago.

DEAR EXAMINER:—In contributing this mite to your pages, remember that I do not claim it as anything new; to your many readers, it is only in confirmation of what has long since been spoken of that I reproduce it, for their careful consideration. Having three cases of this troublesome disorder to report, I will now proceed to do so, if you will pardon me for trespassing on your valuable space.

M. B., a very intelligent woman, applied to me for treatment; her statement was concise, and to the point, replying with great accuracy to all questions asked, rendering it very easy for me to come to a conclusion as to what the matter was. I diagnosed neuralgia of the ovaries. The history of the case I will lay before you, that you may be enabled to follow me, step by step. She told me, for the last two years she experienced great difficulty in menstruating. I examined her, and found considerable fulness in the right illiac region, with great tenderness. Keen, lancinating pains would shoot down the inner part of the thighs, causing her sometimes to cry aloud with pain.

I immediately ordered a plaster of cantharides, to be applied over the seat of pain. After the surface became vessicated, I sprinkled the salts of morphia upon it, at the same time prescribed as follows:—

| | | |
|---|---|---|
| ℞. | Quinia Sulph., | gr. xij. |
| | Ext. Hyoscyam., | gr. xij. |
| | Mice Fiat in Chart., | No. xij. |

I continued to visit her regularly, once a day, for a week, but finding no relief, I resolved to change the treatment. In looking over Braithwaite, part LVIII, page 251, I chanced to see an article on the subject of which I am writing. Although I was at a loss to give its rationale; and never since I have commenced the practice of medicine have I given an article, without being convinced in my own mind what it was going to do. But, however, I resolved to give the remedy proposed a trial. I ordered the following:—

| | | |
|---|---|---|
| ℞. | Ammonia Muriatis, | ʒij. |
| | Tr. Aconiti, | ʒij. |
| | Sy. Aurant. Cort., | ℥viij. |

Mice fiat mistura.

Sig. Teaspoonful three times a day. The second day after having taken the medicine, the sharp pain was gone, she could feel no more of it. I told her to continue the use of the mixture for eight or ten days. She did so; at the end of which time her menses came on, and passed off so easily that she told every one that came in her way. She had a slight return of it, but upon the exhibition of the medicine it entirely disappeared.

Case II. A mulatto woman, aged 29, came to me for relief, having heard from Case I. that I had cured her. I examined her, and found the same symptoms that presented themselves in Case I.

I prescribed the muriate of ammonia and tr. aconiti, in the same doses. At the end of a week she said she was well.

CASE III. An Irish girl was troubled with painful menstruation, and keen, shooting pain in the groin and back. I exhibited the above-named medicines, in the same quantities as in Cases I. and II. She used it for eight or ten days; and at the end of which she pronounced herself well. Furthermore, allow me to say, that this has been over six months ago, and not the slightest return of the disease in either of those cases has appeared.

